# Supplementary material for: Adversarial prompt and fine-tuning attacks threaten medical large language models
Source: Nat Commun. 2025 Oct 9;16:9011. doi: 10.1038/s41467-025-64062-1 (PMC12511276; doi:10.1038/s41467-025-64062-1)
Supplement: Supplementary file 1 — Description of Additional Supplementary File [file 41467_2025_64062_MOESM1_ESM.pdf]

The Description of Additional Supplementary Files

Supplementary Data 1: Attack performance on PubMed Central articles. PE and FT stand for Prompt Engineering and Fine-Tuning respectively. Numbers in the bracket indicate 95% CI, calculated using bootstrapping.

Supplementary Data 2: Attack performance of GPT-4o on full length MIMIC-III patient notes. PE and FT stand for Prompt Engineering and Fine-Tuning respectively. Numbers in the bracket indicate 95% CI, calculated using bootstrapping.
