# Supplementary material for: Adversarial prompt and fine-tuning attacks threaten medical large language models
Source: Nat Commun. 2025 Oct 9;16:9011. doi: 10.1038/s41467-025-64062-1 (PMC12511276; doi:10.1038/s41467-025-64062-1)
Supplement: Supplementary file 4 — Reporting Summary [file 41467_2025_64062_MOESM4_ESM.pdf]

nature portfolio

Corresponding author(s): Zhiyong Lu
Last updated by author(s): Jul 23, 2025

Reporting Summary

Nature Portfolio wishes to improve the reproducibility of the work that we publish. This form provides structure for consistency and transparency in reporting. For further information on Nature Portfolio policies, see our Editorial Policies and the Editorial Policy Checklist.

Statistics

For all statistical analyses, confirm that the following items are present in the figure legend, table legend, main text, or Methods section.

n/a	Confirmed	
☐	☒	The exact sample size (*n*) for each experimental group/condition, given as a discrete number and unit of measurement
☐	☒	A statement on whether measurements were taken from distinct samples or whether the same sample was measured repeatedly
☐	☒	The statistical test(s) used AND whether they are one- or two-sided<br>*Only common tests should be described solely by name; describe more complex techniques in the Methods section.*
☒	☐	A description of all covariates tested
☒	☐	A description of any assumptions or corrections, such as tests of normality and adjustment for multiple comparisons
☐	☒	A full description of the statistical parameters including central tendency (e.g. means) or other basic estimates (e.g. regression coefficient) AND variation (e.g. standard deviation) or associated estimates of uncertainty (e.g. confidence intervals)
☒	☐	For null hypothesis testing, the test statistic (e.g. *F*, *t*, *r*) with confidence intervals, effect sizes, degrees of freedom and *P* value noted<br>*Give P values as exact values whenever suitable.*
☒	☐	For Bayesian analysis, information on the choice of priors and Markov chain Monte Carlo settings
☒	☐	For hierarchical and complex designs, identification of the appropriate level for tests and full reporting of outcomes
☒	☐	Estimates of effect sizes (e.g. Cohen's *d*, Pearson's *r*), indicating how they were calculated

Our web collection on statistics for biologists contains articles on many of the points above.

Software and code

Policy information about availability of computer code

Data collection	We use public datasets including MIMIC-III and PMC-Patients.
Data analysis	We have uploaded all codes to the repository listed in the manuscript.

For manuscripts utilizing custom algorithms or software that are central to the research but not yet described in published literature, software must be made available to editors and reviewers. We strongly encourage code deposition in a community repository (e.g. GitHub). See the Nature Portfolio guidelines for submitting code & software for further information.

Data

Policy information about availability of data

All manuscripts must include a data availability statement. This statement should provide the following information, where applicable:

- Accession codes, unique identifiers, or web links for publicly available datasets
- A description of any restrictions on data availability
- For clinical datasets or third party data, please ensure that the statement adheres to our policy

The MIMIC-III used in this study is available at https://physionet.org/content/mimiciii/1.4/. The PMC-Patients used in this study is publicly available at https://github.com/zhao-zy15/PMC-Patients. Source data are provided with this paper.

Research involving human participants, their data, or biological material

Policy information about studies with human participants or human data. See also policy information about sex, gender (identity/presentation), and sexual orientation and race, ethnicity and racism.

Reporting on sex and gender	Not applicable.
Reporting on race, ethnicity, or other socially relevant groupings	Not applicable.
Population characteristics	Not applicable.
Recruitment	Not applicable.
Ethics oversight	Not applicable.

Note that full information on the approval of the study protocol must also be provided in the manuscript.

Field-specific reporting

Please select the one below that is the best fit for your research. If you are not sure, read the appropriate sections before making your selection.

☐ Life sciences ☒ Behavioural & social sciences ☐ Ecological, evolutionary & environmental sciences

For a reference copy of the document with all sections, see nature.com/documents/nr-reporting-summary-flat.pdf

Behavioural & social sciences study design

All studies must disclose on these points even when the disclosure is negative.

Study description	This study aims to investigate the susceptibility of Large Language Models (LLMs) to adversarial attacks within the context of healthcare applications, specifically medical diagnostics, treatment recommendations, and patient care. By assessing the vulnerability of LLMs against two types of adversarial attacks across three medical tasks, the study evaluates the impact of such manipulations on the models' performance and explores potential defensive mechanisms.
Research sample	The research utilizes two distinct datasets:<br><br>MIMIC-III Dataset: A public database containing de-identified health data from over 40,000 patients at Beth Israel Deaconess Medical Center's critical care units. For the purposes of this study, 1,200 discharge notes are used.<br><br>PMC-Patients Dataset: This dataset consists of 167,000 patient summaries extracted from PubMed Central articles. The first 200 articles from the last 1% of this dataset serve as a test set to evaluate transfer performance for the attack methods.
Sampling strategy	For the MIMIC-III dataset, the study samples 1,200 discharge notes, using the first 1,000 as a training set and the remaining 200 as a test set. For the PMC-Patients dataset, 200 summaries are collected to test transferability and generalization of the findings.
Data collection	Data collection involved summarizing MIMIC-III medical discharge notes using GPT-4 to reduce their length from an average of 4,042 tokens to 696 tokens, accommodating the operational capacity of the models used.
Timing	Not applicable.
Data exclusions	We used the latest of PMC-Patients (most recent PubMed articles) to minimize the influence of pre-training. We used MIMIN-III notes that are longer than 200 characters (including space and symbols).
Non-participation	Not applicable.
Randomization	Not applicable.

Reporting for specific materials, systems and methods

We require information from authors about some types of materials, experimental systems and methods used in many studies. Here, indicate whether each material, system or method listed is relevant to your study. If you are not sure if a list item applies to your research, read the appropriate section before selecting a response.

Materials & experimental systems

n/a	Involved in the study
☒	☐ Antibodies
☒	☐ Eukaryotic cell lines
☒	☐ Palaeontology and archaeology
☒	☐ Animals and other organisms
☒	☐ Clinical data
☒	☐ Dual use research of concern
☒	☐ Plants

Methods

n/a	Involved in the study
☒	☐ ChIP-seq
☒	☐ Flow cytometry
☒	☐ MRI-based neuroimaging

Plants

Seed stocks	Not applicable.
Novel plant genotypes	Not applicable.
Authentication	Not applicable.
